# Integration of palliative care in the management of oral squamous cell carcinoma

**DOI:** 10.6026/97320630019001

**Published:** 2023-01-31

**Authors:** Republica Sridhar, Huwait Etimad, Peter Natesan Pushparaj, Gauthaman Kalamegam

**Affiliations:** 1 RMD Specialities Hospital and RMD Academy for Health, A Unit of RMD Pain and Palliative Care Trust, Chennai, India; 2 King Abdulaziz Univ, Fac Sci, Dept Biochem, Jeddah 21589, Saudi Arabia; 3 King Abdulaziz Univ, King Fahad Med Res Ctr, Expt Biochem Unit, Cell Culture Lab, Jeddah 22252, Saudi Arabia; 4 Center of Excellence in Genomic Medicine Research, and Department of Medical Laboratory Technology, Faculty of Applied Medical Sciences King Abdulaziz University, Jeddah, Saudi Arabia, Jeddah 22252; 5 Center for Transdisciplinary Research, Department of Pharmacology, Saveetha Dental College and Hospitals, Saveetha Institute of Medical and Technical Sciences, Chennai 600077, India.; 6 Pharmaceutical Division, Nibblen Life Sciences Private Limited, Chennai 600061, India

**Keywords:** Oral Squamous cell Cancers, Standard oncological care, Palliative Care, Multi-disciplinary team, Holistic care

## Abstract

Oral Squamous cell Cancers (OSCC) is strongly associated with tobacco consumption. We here in present a case study of a OSCC patient who refused standard oncological care (SOC), to highlight the importance of integrating palliative care (PC) for improved patient outcomes. A 61 years male patient, with history of chewing tobacco for more than 20 years and diagnosed to have OSCC for 1.5 years presented with severe anaemia and a cauliflower-like growth (12 x 10 cm) in the left oral cavity and cheek with greenish-yellow discharge. Pus culture was positive for *K. pneumoniae* and *P. aeruginosa*. Patient is also a known hypertensive for 15 years and a diabetic for 7 years on allopathic treatment. However, the patient refused SOC for oral cancer and relied on siddha treatment. Packed cell transfusions were given to correct anaemia and the blood glucose levels was kept under control. Frequent wound debridement, oral care, antibiotics, balanced-diet and hydration improved wound-bed granulation. Patient and family members were counselled and explained in detail on the need for SOC by sharing previous OSCC patients’ care and outcomes at our centre. Patient gained trust and courage and agreed for chemotherapy, which reduced the disease burden and improved the quality of life (QoL) considerably. Therefore, PC integration at an early stage of treatment is imperative as it reduced (i) the burden of secondary infection, (ii) pain and distress, and (iii) improved the QoL.

## Background:

About 90% of cancers of the oral mucosa are oral squamous cell carcinomas (OSCC) [1]. Cancers of the oral cavity and oropharynx can occur in the tongue, lips, tonsils, and salivary glands, floor of the mouth, gums, or other parts. The risk of developing oral cancer is twice as high in men as in women and as higher in underdeveloped countries than in developed countries. About two-thirds of the world's oral cancers (estimated at 40000 per year) occur in Asian countries. These mainly include India, Sri Lanka, Pakistan, Bangladesh and Indonesia [2]. OSCC is the 16th most common cancer worldwide and the 11th most common in men. [3]. Use of tobacco is a strong risk factor in OSCC and chronic smokers (cigarette/shisha) are more vulnerable to develop oral cancer. Use of tobacco products in other forms such as chewing tobacco or snuff and also chewing of betel quid (containing mainly areca nut and slaked lime) can cause irritation of the lining mucosal membranes leading to dysplasia, with eventual development of oral cancer. Other causes include alcohol intake, human papilloma virus (HPV) infections, inherited or acquired gene mutations and poor nutrition [4]. In addition, abnormalities in the oral microbiome (bacterial dysbiosis) that are usually associated with gingivitis, dental caries and endodontic abscesses are associated with increased risk of OSCC [5]. Importantly, most oral cavity cancers can be prevented if the major risk factors namely tobacco and alcohol which contribute to about 90% of OSCCs are avoided. Both primary prevention which involves avoidance of risk, improving nutrition, personal oral hygiene and HPV vaccination as well the secondary prevention which involves early screening and early detection will help halt the disease progression thus improving survival outcome [6]. Unfortunately, most cases are diagnosed at a later stage, given the latent symptoms in OSCC [7]. Those cancer patients who report with extensive invasion and metastasis and not responding to adjuvant chemo/radiotherapy have poor survival rates. There is an additional subset of cancer patients who are reluctant to accept the available treatment options which can help reduce their disease burden and improve life. This is mainly due to their fears and beliefs about the side effects of chemotherapy/radiotherapy or the surgical outcomes. As a result, they either resort to alternative therapies or just accept the disease process as their fate. In this juncture it is very important to understand the role of multi-disciplinary care, which is the essence of palliative care. It is generally misconceived that palliative care is an 'End-of-Life care'. On the contrary, recent studies have proven that instituting palliative care at a much early stage, right form the diagnosis of cancer have led to remarkable outcome in terms of symptoms control, and overall QoL for both patient and family members/caregivers [8]. In the same context we herein present a case of an OSCC patient who's QoL improved significantly following institution of palliative care.

## Ethical approval:

This case study was approved by the Institutional Ethical Committee vide approval number 2022/RAH001. Informed patient consent was also obtained for this study report.

## Case details:

A 61 years old male, presented with a history of lump in the left side of the oral cavity for 1.5 years and diagnosed as OSCC. Patient is a known diabetic for 7 years on anti-glycaemic therapy with a combination of sulfonylurea, biguanide and alpha-glucosidase inhibitor. He is also a known hypertensive for 15 years on anti-hypertensive therapy with an angiotensin receptor blocker (telmisartan). Patient has no history of smoking/drinking alcohol but has a history of tobacco chewing for more than 20 years. Despite being informed of the presence of OSCC, and the possible treatment options and outcomes, the patient had refused standard oncological care (SOC) given his fears of the side effects associated with chemotherapy/radiotherapy and surgery. Instead, the patient resorted to siddha therapy and had come to our RMD specialities hospital which specializes in pain and palliative care services, with complaints of opening of the mouth, difficulty in swallowing, and pus discharge from the wound in the left cheek and foul odour in mouth.

## Clinical examination:

**Figure 1:**
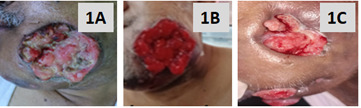
Patient with oral squamous cell carcinoma (OSCC). A: Gross image of the tumour (OSCC) over left cheek which is ulcerated, fungated and having greenish odorous discharge; B: The OSCC ulcer showing healthy granulation tissue following twice a day debridement and treatment with normal saline, metronidazole and betadine solution; C: The healing OSCC ulcer following chemotherapy with paclitaxel, cisplatin and 5-flurouracil.

Clinical examination of the patient revealed severe anaemia associated with breathlessness on mild exertion. The cardiovascular, respiratory, gastro-intestinal, urogenital and skeletal system was clinically normal. Left cheek had an ulcerative mass with a cauliflower like growth (approximately 12 cm x 10 cm in size) which was badly infected and having a greenish-yellow discharge (Figure 1A). Oral hygiene was very poor and halitosiswas present. Tumour involvement extended to retromolar trigone, floor of the mouth and an oro-cutaneous fistula was present. A cervical lymph node enlargement on the left neck was present.

## Investigations:

Complete blood count; fasting and post-prandial blood glucose; serum electrolytes; liver function tests, pus culture and sensitivity were done. Patient was not willing for other investigations (computed tomography and the magnetic resonance imaging) to screen for the extent of the tumour involvement and the presence/absence of any secondaries. The patient was also not willing for biopsy of the tumor to estimate the nature and stage of the disease. Haemoglobin was decreased. And the total count, urea, creatinine, blood glucose values were all increased (Table 1). Serum electrolytes and liver function tests were normal. Pus culture demonstrated the presence of *Klebsiella pneumoniae* and *Pseudomonas aeruginosa*.

## Treatment:

**Figure 2:**
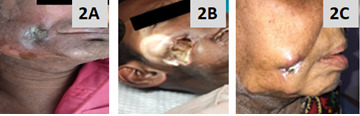
Gross images showing healing ulcers in few other OSCC patients following treatment with chemotherapy in our hospital.

Upon admission, the patient was given two units of packed cell transfusion. Under sterile conditions wound debridement and cleaning with metronidazole, betadine and normal saline solutions was done twice a day. Antibiotics (linezolid, meropenem) were initiated following pus culture and sensitivity from the wound site. Oral care by the dentist was provided which involved debridement and removal of the slough from inside of the oral cavity followed by washes with metronidazole, betadine and normal saline twice a day. Nutritional care with balanced diet and adequate hydration was provided. Patient was also given his regular medications for hypertension and diabetes, with injectable insulin as and when required to control the glucose levels. Patient's general health and biochemical parameters significantly improved. The hemogram and the biochemical parameters gradually returned to near normal levels (Table 1). Continued wound debridement and care led to the development of healthy granulation tissue (Figure 1B). We the palliative care team apart from providing an all-inclusive care, (general patient care, pain care, dental care, nutritional care and also accommodating the patient believed siddha care) regularly communicated with the patient and his family members, explaining in detail about the need for adjuvant chemotherapy followed by surgery. We were steadfast in counselling the patient regarding his current health status, the possible outcomes and the need for optimal oncological care. We also continued our communications with the care-givers, and family members and reinforced that standard oncological care will help in reducing the disease burden. We further shared necessary details of similar patients treated under our palliative care team and their images of improvementfollowing treatment (Figure 2A-C). This largely helped the patient to understand the importance of standard oncological care, as well as to gain trust and courage. The patient finally agreed to undergo chemotherapy and a cycle with paclitaxel, cisplatin and 5-flurouracil was instituted. Subsequently, the patient returned to his native and underwent the second cycle of chemotherapy followed by radiation. Given his belief, the patient still continues to take siddha treatment. It is important to understand that that integrated care has significantly reduced the tumour burden (Figure 1C) and associated distress.

## Discussion:

In general, the early OSCC constitutes a favourable subgroup among the head and neck squamous cell carcinomas. Tobacco consumption in any form (smoke, oral, snuff, dissolved) is well established to be associated with oral cancer. Consumption of alcohol, chewing betel quid, and poor oral hygiene also predispose to OSCC. Furthermore, the linkage of oral microbiota with cancer is vast emerging. Patients suffering from gum and periodontal diseases (gingivitis, periodontitis) caused by pathogenic bacteria have a two to five times higher risk of cancer causation, compared to healthy individuals. Cancer induction by microbial pathogens can be due to inhibition of apoptosis, activation of cell proliferation, promotion of cell invasion and chronic inflammation [9,10]. Surgery is the ideal management for OSCC, and carries a much favourable outcome with reduced treatment induced morbidity. Despite this fact, there remain a subset of patients who are reluctant to undergo standard oncological care, which is mainly to avoid the undesirous side effects associated with cancer therapeutics. Hence there is a tremendous need to explain both the patient and the family members/care givers about various issues including (i) the nature of the disease, (ii) existing treatment options, (iii) possible outcomes, (iv) managing pain and debilitating symptoms, (v) methods to reduce the disease burden, (vi) improving the QoL, (vii) ending the life with dignity, (viii) whether end management to be in hospital or home, and (ix) whether resuscitation is needed or not. Although, the standard oncological care team can handle most of the treatment related issues, there still remain a lot of areas which can be effectively handled by a palliative care team. As such, all clinical decisions with regards to the management of early OSCC should ideally be taken by a multi-disciplinary palliative care team experienced in the care of such cases. Cooperation between surgeons, radiation oncologists, dentists, palliative care specialists, nurses and speech therapists is important for holistic patient care with good outcomes [11]. Patients, who are averse to conventional treatment as noted in our case study, if not cared holistically, may gradually become withdrawn from the family as well as society. Their disease burden, economic burden and psycho-social issues can severely affect the QoL. Palliative care encompasses a multi-disciplinary approach as demonstrated in our case study, wherein use of alternative (siddha) medicine which patient strongly believed in was also integrated along with conventional cancer treatment. Lifestyle modifications and integration of mind and body therapies such as acupuncture, massage, meditation, yoga and natural therapy is also reported to help in relieving pain and distress and improve QoL [12].

## Conclusions:

Standard oncologic treatment remains the cornerstone of therapy for early-stage OSCC with favourable outcomes. Cancer patients have different attitudes toward conventional cancer therapies and seek alternative therapies based on their beliefs. The integration of palliative care in early stages will help to fill the existing gaps and achieve optimal outcomes. Multidisciplinary care has improved the overall health and attitude of the OSCC patient, as well as the response of caregivers in managing the disease, underscoring the need for early integration of palliative care into patient care.

## Figures and Tables

**Figure 3:**
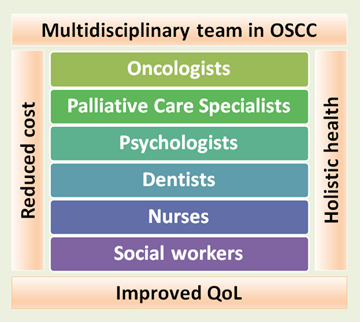
Diagrammatic representation of the multi-disciplinary team which is an essential component of palliative care for providing holistic health. Current guidelines strongly advocate the integration of the multi-disciplinary team in regular clinical/hospital setting for enhanced health care.

**Table 1:** The haematological and biochemical values at admission and following treatment

Biochemical Parameters	Values at admission	Values at discharge
Haemoglobin (gm/dl)	7	11
Total WBC count (cells/cu mm)	28100	12300
Blood sugar (mg /dl)		
Fasting	152	104
Post-prandial	240	194
Random		88 to 186
HbA1C (%)	8.4	8
Urea (mg /dl)	66	27
Creatinine (mg /dl)	1.6	1
